# Association between placental malaria, postnatal linear growth, and body mass index in the Dogon Longitudinal Study, Mali

**DOI:** 10.1186/s12936-025-05776-x

**Published:** 2026-01-07

**Authors:** Chloe Merritt, Claudius Vincenz, Zachary Dolo, Kerby Shedden, Beverly I. Strassmann

**Affiliations:** 1 Department of Anthropology, University of Michigan, 101 West Hall, 1085 South University Avenue, Ann Arbor, MI 48109-1107 USA; 2 Research Center for Group Dynamics, Institute for Social Research, University of Michigan, Ann Arbor, MI USA; 3 Independent Investigator, Bandiagara Cercle, Mali; 4 Department of Statistics, University of Michigan, Ann Arbor, MI USA

**Keywords:** Placental malaria, Malaria, Postnatal growth, Linear growth, BMI, Sub-Saharan Africa

## Abstract

**Background:**

Malaria is a primary cause of morbidity and mortality in Mali, with women at high risk for malaria infection during pregnancy. Placental malaria (PM) has been linked to adverse neonatal outcomes such as low birth weight, decreased birth length, and decreased placental weight, but few studies have tested for associations between PM and postnatal growth. This study examined the relationship between PM and linear growth, weight, and body mass index (BMI) from birth to age 5 years.

**Methods:**

The study participants (N = 317) were members of the F2 generation of the Dogon Longitudinal Study, a multigenerational prospective cohort study conducted in the District of Bamako and on the Bandiagara Escarpment in central Mali. Placental samples were collected for each participant and evaluated by histology to determine PM infection stage and parasite density. Participant’s height, weight, and BMI were measured approximately twice per year from birth until a median age of 6.8 years (maximum age 12.3 years). Linear mixed models were used to investigate the relationship between PM and height, weight, and BMI from birth to age 5 years.

**Results:**

Linear growth in height at age 6 months was lower in infants from placentas with severe parasite density compared to infants from placentas in which no parasites were detected (−0.83 cm; 95% CI −1.63, − 0.03; p = 0.042), with a trend of decreased height continuing to age 5 years. Severe parasite density was also associated with a statistically significant increase in BMI at 2 years (0.56 kg/m^2^; 95% CI 0.13, 1.00; p = 0.011), 3 years (0.82 kg/m^2^; 95% CI 0.35, 1.30; p = 0.001), 4 years (1.09 kg/m^2^; 95% CI 0.53, 1.65; p > 0.001), and 5 years (1.35 kg/m^2^; 95% CI 0.68, 2.01; p > 0.001) of age when controlling for birth weight. No statistically significant associations were observed between parasite density and weight.

**Conclusions:**

Severe malaria parasite density in the placenta was associated with birth length, as shown in previous studies, and, in addition, was associated with decreased linear growth to age 5 years. Moreover, this study provided the first evidence that PM is associated with increased postnatal BMI.

**Supplementary Information:**

The online version contains supplementary material available at 10.1186/s12936-025-05776-x.

## Background

In 33 countries in sub-Saharan Africa in 2023, approximately 12.4 million women experienced malaria in pregnancy (representing 34% of total estimated pregnancies) and delivered 901,000 low birthweight neonates [1]. Erythrocytes infected with mature asexual *Plasmodium falciparum* parasites may sequester in the placenta during pregnancy [2]. Placental malaria (PM) is defined by the presence of infected erythrocytes or haemozoin in the placental intervillous space and is associated with maternal illness and anaemia [3], as well as increased risk of maternal death before and after childbirth [1]. PM is also associated with preterm birth [1, 4] as well as low birth weight and birth length [1, 2].

Although abundant evidence links PM to adverse neonatal outcomes, there is limited evidence regarding the association between PM and postnatal growth. In the Gambia, PM was negatively associated with infant’s weight from birth to 12 months, independent of low birth weight [5]. In Malawi, placental or peripheral malaria at delivery was associated with low weight-for-age at 12 months, independent of low birthweight [6]. Similarly in southwestern Uganda, PM was associated with decreased weight and linear growth at 12 months [7]. To our knowledge, no studies have investigated the possibility of an association between PM and postnatal growth after age 12 months. Expanding the time-frame for postnatal follow-up of children from gestations with PM is needed since linear growth retardation and stunting are associated with increased morbidity and mortality of offspring [8, 9], elevated risk of metabolic disease in adulthood [8], lower cognitive function [9–11], as well as reduced economic capacity and lower wages [8, 9, 12]. This study investigated the potential associations between PM and linear growth, weight, and BMI in the first five years of postnatal life in Mali, West Africa.

## Methods

### Ethics approval

All data were collected with informed individual consent/assent. This study was approved by the University of Michigan institutional review board (IRB) (HUM00043670) and by the Malian IRB (authorization No. 2016/68/CD/FMPOS) in conjunction with annual approval by the Malian government from 1998 (N◦ 25/CNRST/98) to 2024 (N◦ 2/2024-MESRS/CNRST). Annual ethical approval was also obtained at the community level in Mali.

### Study population and design

The study participants were members of the F2 generation of the Dogon Longitudinal Study, a multigenerational prospective cohort study that was conducted in the District of Bamako and on the Bandiagara Escarpment in central Mali [2, 13]. Enrollment of the F1 participants occurred from 1998 to 2000 at a median (25th, 75th percentiles) age of 1.59 (0.62, 3.44) years [13]. The F2 participants were enrolled at birth from 2011 to 2019 with regular follow-up to a median (25th, 75th percentiles) age of 6.81 (5.60, 7.82) years.

### Placental collections

In Bamako, the placental collections took place at the Centre Hospitalier Universitaire Cabriel Touré and the Centre de Santé de Référence Commune V and VI, among other health facilities. In the villages, the placental collections took place in a single rural hospital that serves over 50 villages on the Bandiagara Escarpment. Approximately one gram of fetal tissue destined for histological examination was dissected from the interior of each of two placental cotyledons. The full protocol for the placental collections is described elsewhere [2]. After the dissections, the placentas were returned to the families. This research did not adversely impact any local customs as the Dogon do not include the placenta in any rites or ceremonies. Services such as antenatal visits, bloodwork at the Institut Merieux, and ultrasounds were offered to women in Bamako. In the villages, only antenatal visits were offered as other services were unavailable.

### Histology

Details about specimen preparation and evaluation of histology have been previously described [2]. A placental pathologist and research assistant performed blinded histological evaluations using established guidelines for the assessment of PM [14, 15]. Slides were scored for malaria parasites (presence and density), haemozoin pigment (presence and density), and malaria infection stage (none, acute, chronic, past).

### Definitions

Parasite density was based on histological slides and was defined by the density of infected maternal erythrocytes (parasitaemia) in the interstitial space of the placenta and was classified as not present, mild (< 1% of maternal erythrocytes infected), moderate (1–10% of maternal erythrocytes infected), or severe (> 10% of maternal erythrocytes infected in 20–40 fields at 40–100X magnification). Gravidity was classified as primigravida for a mother’s first pregnancy or multigravida for a mother’s second or later pregnancies. Perinatal mortality was defined as stillbirths and deaths within the first 7 days of life.

### Height, weight, body mass index

Anthropometric data for the F2 children were obtained by midwives at birth and by the research team on each follow-up visit. Recumbent length (age < 2 years) or postnatal height (age ≥ 2 years) was measured in triplicate to the nearest 0.1 cm using a portable infantometer or stadiometer (Perspective Enterprises) and the three values were averaged. Postnatal weight was measured to the nearest 0.1 kg using battery powered electronic scales (Tanita & Seca). Body mass index (BMI) was calculated as weight (kg) divided by height squared (m^2^). Maternal pre-pregnancy BMI was defined as the last BMI measurement before the estimated date of conception, which was back-calculated as nine months before the date of birth of the F2 child.

### Statistical analysis

Statistical analysis was performed in R version 4.3.3 using the packages lme4 and splines to construct linear mixed models with cubic regression splines. Height, weight, and BMI were modeled separately as dependent variables using only measurements taken from birth to age 5 years. Models were fit with random slopes and intercepts that accounted for repeated measurements and clustering by mother, as 40.4% of study participants had at least one maternal sibling in the study. Among possible random slope and intercept combinations, final model choice was based on AIC. The height and weight models included uncorrelated random age slopes and random intercepts for study participant and for mother. A cubic B-spline basis with five degrees of freedom was included in the height and weight models to capture the nonlinear association between age and either height or weight. In the BMI model, a cubic B-spline basis was included with knots at birth, 6 months, 1 year, and 5 years to capture the nonlinear association between age and BMI. The BMI model included correlated random age slopes and random intercepts for study participant and for mother. All models included an interaction between participant’s age and parasite density and controlled for maternal covariates: gravidity, height, education, pre-pregnancy BMI, and residence during pregnancy (the city of Bamako or rural villages). Maternal age was not included as a covariate as it was highly correlated with gravidity (r^2^ = 0.53, p < 0.001). Sex-combined and sex-stratified models were created to investigate general and sex specific effects. Sex-combined models included an interaction between sex and cubic B-spline basis to account for sex differences in growth trajectories. Contrast tests were used to test for associations between parasite density and height, weight, or BMI from birth to age 5 years. P-values < 0.05 were considered significant.

## Results

### Study participant characteristics

Descriptive statistics for children (N = 317) and their mothers are shown in Table 1. Eighty-four percent of mothers lived in rural villages on the Bandiagara escarpment for most of their pregnancies, and the remaining 15% of mothers resided in Bamako. Self-reports for schooling were: none (26%), some primary education (38%), some secondary education (25%), and beyond secondary schooling (11%). Mean (SD) maternal age at delivery was 20.4 ± 2.2 years and mean (SD) pre-pregnancy BMI was 21.9 ± 2.5 kg/m^2^. Mean (SD) maternal height was 158.5 ± 5.7 cm. Fifty-seven percent of mothers were primigravida and approximately 43% were multigravida (32% second, 8% third, 3% fourth, and 1% fifth pregnancies). The mean (SD) birth length was 49.1 ± 1.7 cm, birth weight was 2729 ± 395 g, and BMI at birth was 11.28 ± 1.44 kg/m^2^. Twenty-five percent of neonates had low birth weight (< 2500 g) and 75% were of normal birth weight. Among the 317 placentas analysed for parasite density, the prevalences were: 7% severe (N = 21), 16% mild or moderate (N = 50), 76% no parasites detected (N = 241), and 2% unscorable (N = 5). Tables 2 and 3 show the observed height and BMI, respectively, of participants at the 50th (10th–90th) percentiles from birth to 5 years.

**Table 1:** Maternal, placental malaria, and birth characteristics (N = 317 mother–child pairs)

Categorical variables	Female n (%)	Male n (%)	Total n (%)
Sex	150 (47.3)	167 (52.7)	317 (100)
Mothers			
Residence during pregnancy			
Village	130 (86.7)	138 (82.6)	268 (84.5)
Bamako	20 (13.3)	29 (17.4)	49 (15.5)
Education			
No schooling	40 (26.7)	43 (25.7)	83 (26.2)
Some primary	62 (41.3)	58 (34.7)	120 (37.9)
Some secondary	34 (22.7)	46 (27.5)	80 (25.2)
Beyond secondary	14 (9.3)	20 (12.0)	34 (10.7)
Parity			
Primigravida	79 (52.7)	101 (60.5)	180 (56.8)
Multigravida	71 (47.3)	66 (39.5)	137 (43.2)
Births			
Survival status			
Survived	148 (98.7)	157 (94.0)	305 (96.2)
Perinatal mortality	2 (1.3)	10 (6.0)	12 (3.8)
Birth weight			
> 2500 g	111 (74.0)	126 (75.4)	237 (74.8)
< 2500 g	39 (26.0)	40 (24.0)	79 (24.9)
Missing	0 (0.0)	1 (0.6)	1 (0.3)
Placental malaria			
Parasite density			
None	118 (78.7)	123 (73.7)	241 (76.0)
Mild or Moderate	21 (14.0)	29 (17.4)	50 (15.8)
Severe	9 (6.0)	12 (7.2)	21 (6.6)
Unscorable	2 (1.3)	3 (1.8)	5 (1.6)
Quantitative variables	Female mean (SD)	Male mean (SD)	Total Mean (SD)
Maternal age at delivery (years)	20.55 (2.15)	20.35 (2.18)	20.45 (2.17)
Maternal pre-pregnancy BMI (kg/m^2^)	21.77 (2.38)	21.85 (2.47)	21.82 (2.43)
Maternal height (cm)	158.41 (5.86)	158.50 (5.57)	158.50 (5.70)
Birth length (cm)^a^	49.1 (1.6)	49.2 (1.8)	49.1 (1.7)
Birth weight (g)^a^	2677 (364)	2778 (418)	2729 (395)
Birth BMI (kg/m^2^)^a^	11.10 (1.33)	11.44 (1.52)	11.28 (1.44)

^a^for 305 births excluding stillbirths and deaths in the first seven days.

*BMI* body mass index, *SD* standard deviation

**Table 2:** Observed height (cm) of F2 participants at the 50th (10th–90th) percentiles for specified age ranges

	No parasites detected (N = 241)	Mild or moderate parasite density (N = 50)	Severe parasite density (N = 21)
At birth	49.0 (48.0–51.0)	49.0 (48.0–50.0)	49.0 (46.0–49.3)
Birth to 6 months	59.4 (54.8–63.8)	59.1 (53.9–61.4)	57.3 (53.7–59.8)
6 months to 1 year	69.5 (66.2–73.0)	70.1 (67.4–72.3)	67.9 (65.3–72.2)
1 year to 18 months	75.3 (71.6–79.0)	74.4 (72.5–78.1)	73.2 (70.0–75.9)
18 months to 2 years	80.0 (76.2–82.7)	80.7 (75.9–83.7)	79.6 (74.7–81.4)
2 to 3 years	84.1 (79.9–88.1)	85.4 (80.0–88.3)	84.3 (79.6–86.0)
3 to 4 years	92.6 (87.9–96.6)	91.1 (86.8–97.0)	89.7 (85.3–92.1)
4 to 5 years	99.6 (94.7–105.1)	97.5 (93.5–103.6)	96.1 (92.2–98.2)

**Table 3:** Observed BMI^1^ (kg/m^2^) of F2 participants at the 50th (10th–90th) percentiles for specified age ranges

	No parasites detected (N = 241)	Mild or Moderate parasite density (N = 50)	Severe parasite density (N = 21)
At birth	11.5 (9.8–13.1)	10.6 (9.0–12.4)	10.3 (8.8–11.8)
Birth to 6 months	16.0 (14.5–18.7)	16.1 (13.8–18.8)	16.6 (14.2–17.6)
6 months to 1 year	16.2 (14.4–18.3)	15.9 (14.0–17.5)	16.3 (14.1–17.6)
1 year to 18 months	15.6 (14.2–17.2)	15.4 (13.7–16.6)	15.2 (14.2–17.0)
18 months to 2 years	15.2 (14.0–17.0)	15.5 (14.0–17.1)	15.9 (14.0–16.5)
2 to 3 years	15.4 (14.1–16.7)	15.3 (14.6–16.6)	15.9 (14.5–17.0)
3 to 4 years	15.2 (14.1–16.4)	15.3 (14.6–16.5)	15.9 (14.5–17.3)
4 to 5 years	14.8 (13.5–15.9)	15.1 (14.0–16.0)	15.6 (14.8–15.6)

^*1*^*BMI* body mass index

### Height

**Fig. 1:**
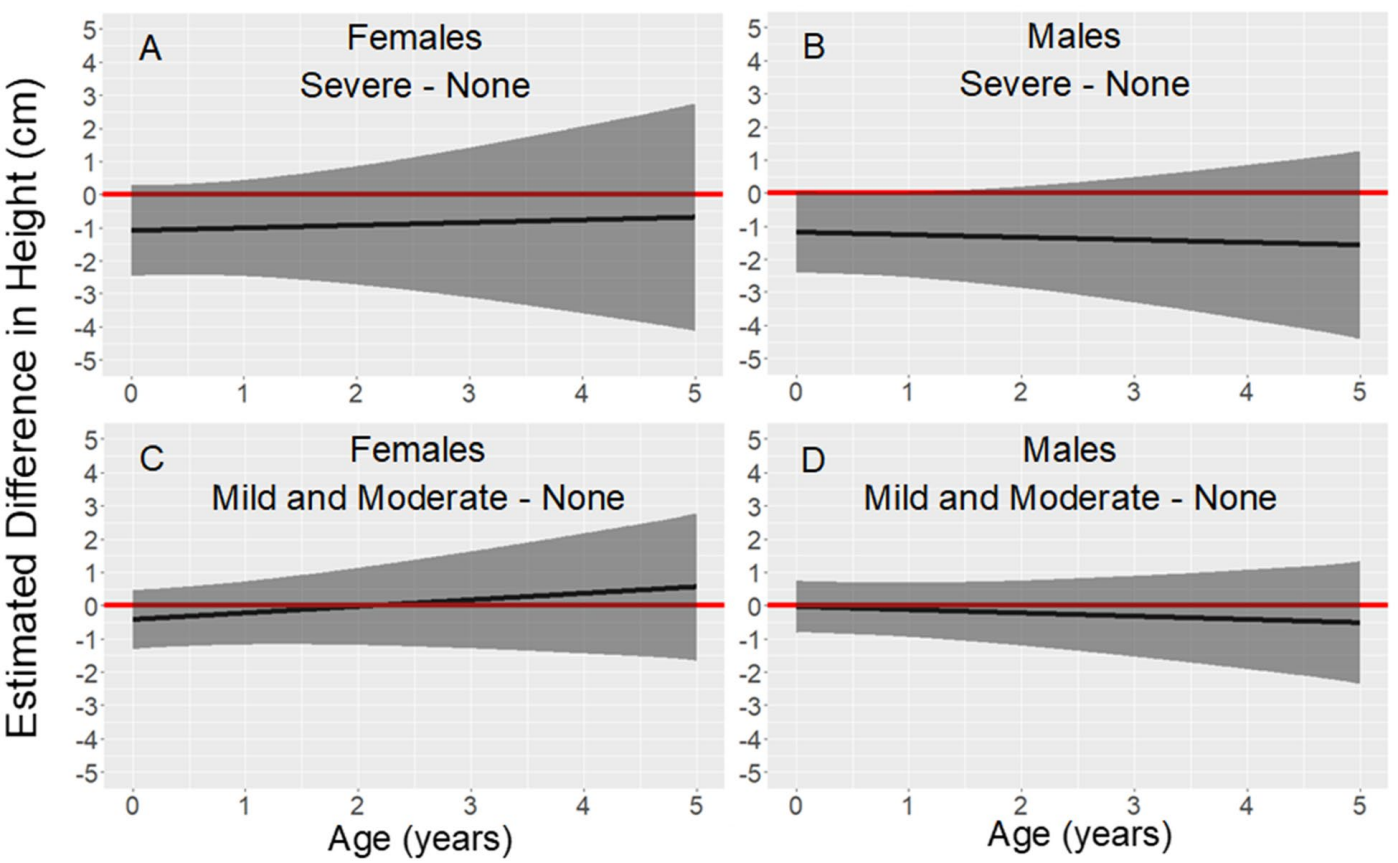
Association between parasite density and height from birth to age 5 years. Legend: Estimated differences in height (solid black line) and 95% pointwise confidence intervals (dark grey regions) from linear mixed models stratified by sex and adjusted for residence during pregnancy, maternal pre-pregnancy BMI, maternal height, maternal education, and gravidity. The red line fixed at zero shows no predicted difference. The number of study participants (N) with at least one height measurement varied by age: birth N = 301, from birth to age 1 year N = 287, from age 1 to 2 years N = 244, from age 2 to 3 years N = 185, from age 3 to 4 years N = 159, and from age 4 to 5 years N = 172

Figure 1 shows the estimated difference in height for each sex, from birth to age 5 years, for children with placentas that had mild/moderate or severe parasite density compared to children with placentas in which no malaria parasites were detected. The models controlled for maternal covariates including residence during pregnancy, pre-pregnancy BMI, height, education, and gravidity.

The model for males (Fig. 1B and D) showed a significant difference in estimated height between children from placentas with severe parasite density compared to children from placentas in which no parasites were detected when analysed at birth (−1.17 cm, p = 0.050), 6 months (−1.21 cm, p = 0.043), and 1 year (−1.24 cm, p = 0.047), with a similar trend at 18 months (−1.28 cm, p = 0.061), 2 years (−1.32 cm, p = 0.084), 3 years (−1.40 cm, p = 0.147), 4 years (−1.48 cm, p = 0.217), and 5 years (−1.55 cm, p = 0.281) of age. Although not statistically significant, the model for females (Fig. 1A and C) showed a trend of decreased height for severe parasite density compared to no parasites detected at birth (−1.02 cm, p = 0.116), 6 months (−0.98 cm, p = 0.134), 1 year (−0.94 cm, p = 0.180), 18 months (−0.89 cm, p = 0.250), 2 years (−0.85 cm, p = 0.334), 3 years (−0.77 cm, p = 0.500), 4 years (−0.69 cm, p = 0.632), and 5 years (−0.60 cm, p = 0.729) of age. The 95% confidence intervals, point estimates, and p-values at yearly intervals from birth to age five years for males and females are shown in Supplementary Table 1, Additional File 1. The model combining males and females (Supplementary Table 2, Additional File 2) showed a significant difference in estimated length between children from placentas with severe parasite density compared to children from placentas with no parasites detected at birth (−0.83 cm, p = 0.041) and 6 months (−0.83 cm, p = 0.042) of age, with a similar trend when height was measured from one to five years of age. No evidence was observed for a difference in height from birth to age five years when comparing mild or moderate parasite density to no parasites detected.

### Weight

**Fig. 2:**
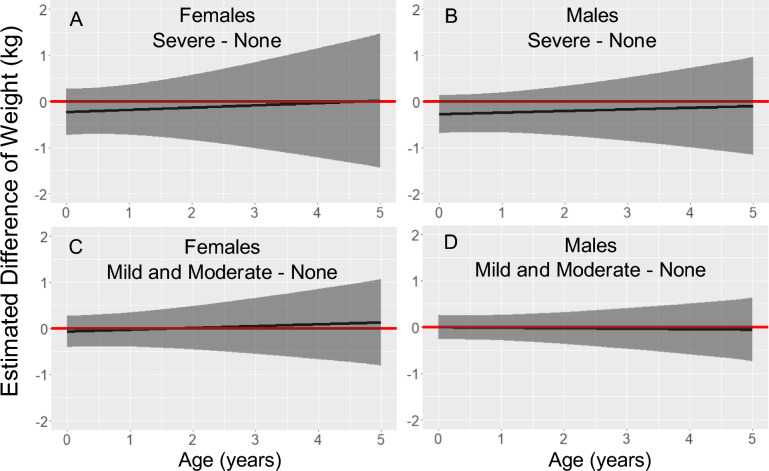
Association between parasite density and weight from birth to age 5 years. Legend: Estimated differences in weight (solid black line) and 95% pointwise confidence intervals (dark grey regions) from linear mixed models stratified by sex and adjusted for residence during pregnancy, maternal pre-pregnancy BMI, maternal height, maternal education, and gravidity. The red line fixed at zero to show no predicted difference. The number of study participants (N) with at least one weight measurement varied by age: birth N = 301, from birth to age 1 year N = 288, from age 1 to 2 years N = 242, from age 2 to 3 years N = 185, from age 3 to 4 years N = 159, and from age 4 to 5 years N = 172

Figure 2 shows the estimated difference in weight for each sex, from birth to age 5 years, for children from placentas with mild/moderate or severe parasite density compared to children from placentas in which no malaria parasites were detected. The models controlled for maternal covariates including residence during pregnancy, pre-pregnancy BMI, height, education, and gravidity.

Neither the model for females (Fig. 2A and C), males (Fig. 2B and D), or the sex-combined model showed evidence for a difference in weight from birth to age 5 years when severe or mild/moderate parasite density was compared to no parasites detected. The 95% confidence intervals, point estimates, and p-values at yearly intervals from birth to age 5 years for males and females are shown in Supplementary Table 3, Additional File 3. Results for males and females combined are shown in Supplementary Table 2, Additional File 2.

### Body mass index

**Fig. 3:**
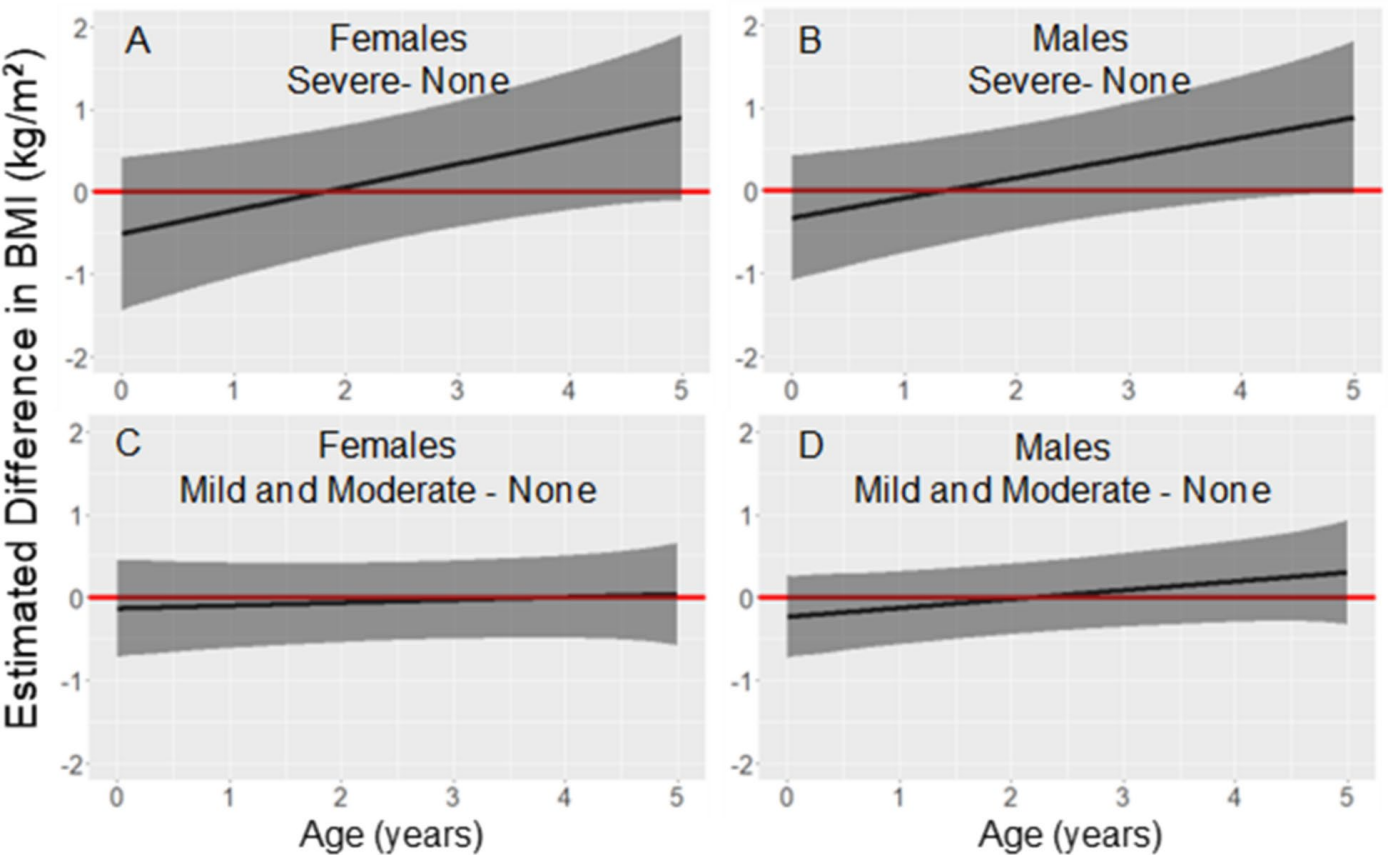
Association between parasite density and BMI from birth to age 5 years. Legend: Estimated difference in BMI (solid black line) and 95% pointwise confidence intervals (dark grey regions) from linear mixed models stratified by sex and adjusted for maternal pre-pregnancy BMI, maternal height, gravidity, maternal education, and residence during pregnancy. The red line is fixed at zero to show no predicted difference. The number of study participants (N) with at least one BMI measurement (N) varied by age: birth N = 301, from birth to age 1 year N = 287, from age 1 to 2 years N = 242, from age 2 to 3 years N = 185, from age 3 to 4 years N = 159, and from age 4 to 5 years N = 172

Figure 3 shows the estimated difference in BMI for each sex, from birth to age 5 years, for children from placentas with mild/moderate or severe parasite density compared to children from placentas in which no malaria parasites were detected. The models controlled for maternal covariates including residence during pregnancy, pre-pregnancy BMI, height, education, and gravidity.

In the models for both males (Fig. 3B and D) and females (Fig. 3A and C), the estimated BMI increased with age when placental parasite density was severe compared to no parasites detected. By age 5 years the estimated difference was positive and significant for males (0.89 kg/m^2^, p = 0.041) and nearly significant for females (0.90 kg/m^2^, p = 0.064). At birth the pattern was the opposite as neonates had lower BMIs when parasite density was severe (−0.33 kg/m^2^, p = 0.374 for males; −0.51 kg/m^2^, p = 0.277 for females). For males and females from placentas with severe parasite density, mean predicted BMI shifted from low at birth (11.19 kg/m^2^, BMI for age z-score = −1.94, for males; 10.37 kg/m^2^, BMI for age z-score = −2.73, for females) to a normal BMI at age 5 years (15.00 kg/m^2^, BMI for age z-score = −0.15, for males; 14.59 kg/m^2^, BMI for age z-score = −0.47, for females), according to the World Health Organization (WHO) child growth standards [16]. There was no evidence for a difference in BMI from birth to age 5 years when mild/moderate parasite density was compared to no parasites detected. The 95% confidence intervals, point estimates, and p-values at yearly intervals from birth to age 5 years for males and females are shown in Supplementary Table 4, Additional File 4.

The model combining males and females (Supplementary Table 2, Additional File 2) showed a significant difference in estimated BMI at age 4 years (0.62 kg/m^2^, p = 0.022) and age 5 years (0.87 kg/m^2^, p = 0.006). When controlling for birth weight, the association between severe parasite density and estimated difference in BMI was significant at 2 years (0.56 kg/m^2^, p = 0.011), 3 years (0.82 kg/m^2^, p = 0.001), 4 years (1.09 kg/m^2^, p > 0.001), and 5 years (1.35 kg/m^2^, p > 0.001) of age. The model combining males and females that controlled for BMI at birth showed a significant estimated difference at ages 3 to 5 years as well. In all sex-combined models, neonates had lower predicted BMIs when parasite density in the placenta was severe. Results of sex-combined models controlling for birth weight and birth BMI are shown in Supplementary Table 5, Additional File 5.

## Discussion

### Height

In the Dogon of Mali, severe parasite density in the placenta was associated with lower predicted recumbent length in males from birth to 12 months when compared to males from placentas with no detected parasites. The results for females followed a similar trend. In the Dogon and other populations, birth length was negatively associated with PM [1, 2]. The present findings suggest that this association continued postnatally and expand upon a previous study in Uganda that found PM to be associated with decreased length at 12 months [7]. Furthermore, in the present study, the magnitude of the association between PM and height was relatively consistent in both males and females from 12 months to 5 years of age, implying that the association persists to age 5 years. Gaps in follow-up due to the Covid-19 pandemic reduced the sample size for children after age 2 years, which probably increased the standard errors at older ages.

### Weight

Numerous studies have established a relationship between PM and low birth weight [2, 4], including a previous study of the Dogon [2]. Two other studies, in Uganda and the Gambia, reported that PM was associated with reduced weight at age 12 months [5, 6]. However, the present study found no evidence for a significant association between placental parasite density and child weight at age 12 months. Weight is subject to variability from short-term exposures such as illness or food intake. Furthermore, day-to-day fluctuations in weight in children aged one to three years may vary by up to 300 g from their underlying weight trajectories [17]. The variability in weight from short-term exposures and day-to-day fluctuations may have increased the standard errors of the observed weight differences.

### Body mass index

Previous studies, in Malawi and the Gambia, reported that PM was negatively associated with either BMI or weight-for-length, respectively, at age 12 months [5, 6]. The present study in Mali found no evidence for a significant association between placental parasite density and postnatal BMI at age 12 months. However, in contrast with previous findings, when birth weight was controlled, children from placentas with severe parasite densities had higher BMIs than did their counterparts from normal placentas. This association was significant from age 2 to 5 years. For children from placentas with severe parasite density, the mean predicted BMI at birth was low (BMI-for-age z-score approximately −2) according to WHO child growth standards [16]. However, by age 5 years, the mean predicted BMI of these children was in the normal range (BMI-for-age z-score approximately 0). A similar trend was observed when birth BMI was controlled and when analysing males and females separately. These findings indicate that the associations between PM and BMI persisted beyond 12 months of age, shifting from a negative association at birth to a positive association in early childhood.

The novel finding that PM was associated with increased BMI but not weight in early childhood warrants further investigation. One question is to what extent was the increased BMI caused by decreased height? Or was the increased BMI caused in part by metabolic programming that favored energy storage? Children who experienced fetal growth restriction (FGR) often experience catch-up growth in the first years of life. In some cases, there is strong gain in fat mass instead of muscle or height, causing increased BMI [18]. Children who experience rapid BMI increases after FGR are at risk for insulin resistance, central obesity, and metabolic syndrome later in childhood or adulthood [19]. In the present study, the association between PM and postnatal BMI emerged more clearly when birth weight was controlled, suggesting that the increase in BMI did not result entirely from postnatal catch-up following low birth weight. Future research should address the possibility that PM may be another pathway contributing to metabolic syndrome.

### Limitations

This study is limited by the lack of data on gestational age at delivery. Moreover, a larger sample size would have been beneficial for detecting associations after age 2 years. The results pertain to this Dogon cohort and should not be construed as generalizable to other populations or settings. Furthermore, as this was an observational study, causation between placental parasite density and postnatal growth cannot be inferred.

## Conclusion

In this prospective cohort study of Dogon children in Mali, children from placentas with severe malaria (*Plasmodium falciparum*) parasite density were shorter relative to children from placentas without detected malaria infection. In males, the deficits in height increased consistently with age from birth to age 5 years. The magnitude of these deficits was −1.2 cm at birth and −1.6 cm at age 5 years. Perhaps due to small sample sizes at older ages, deficits were statistically significant (p < 0.05) only at birth, age 6 months, and age 12 months. Females from placentas with severe parasite density were also shorter than their counterparts from healthy placentas, but the difference was not statistically significant at any age. At birth, males and females from placentas with severe parasite density were predicted to have a mean BMI deficit of −0.53 kg/m^2^ and −0.33 kg/m^2^, respectively, although these deficits were not statistically significant. By 5 years of age, mean predicted BMI was 0.88 kg/m^2^ higher for both males and females whose placentas had severe parasite density, and was statistically significant for males. This change represented recovery from low BMI at birth (BMI-for-age z-score < −2) to normal BMI (BMI-for-age z-score approximately 0) at age 5 years. Since BMI tracks from childhood to adulthood [20, 21], this novel result suggests the possibility that PM may predispose children for higher BMI, which could have downstream implications for adult health. Future longitudinal research should further explore associations between PM and BMI during postnatal life.

## Supplementary Information

Supplementary material 1. Table of the 95% confidence intervals, point estimates, and p-values of association between parasite density and heightat yearly intervals from birth to age five years stratified by sex. 

Supplementary material 2. Table of the 95% confidence intervals, point estimates, and p-values of association between parasite density and height, weight, and BMIat yearly intervals from birth to age five years. 

Supplementary material 3. Table of the 95% confidence intervals, point estimates, and p-values of association between parasite density and weightat yearly intervals from birth to age five years stratified by sex. 

Supplementary material 4. Table of the 95% confidence intervals, point estimates, and p-values of association between parasite density and BMIat yearly intervals from birth to age five years stratified by sex. 

Supplementary material 5. Table of the 95% confidence intervals, point estimates, and p-values of association between parasite density and BMIat yearly intervals from birth to age five years adjusting for birth weight and birth BMI. 

## Data Availability

The dataset generated and analysed during this study will be made available at ICPSR at the University of Michigan.

## Abbreviations

PM: Placental malaria
BMI: Body mass index
