# Supplementary material for: Association between placental malaria, postnatal linear growth, and body mass index in the Dogon Longitudinal Study, Mali
Source: Malar J. 2026 Jan 7;25:78. doi: 10.1186/s12936-025-05776-x (PMC12870135; doi:10.1186/s12936-025-05776-x)
Supplement: Supplementary file 2 — Supplementary material 2. Table of the 95% confidence intervals, point estimates, and p-values of association between parasite density and height, weight, and BMIat yearly intervals from birth to age five years. [file 12936_2025_5776_MOESM2_ESM.docx]

|  | | | | Height (cm) | | | |  | Weight (kg) | | | |  | BMI (kg/m^2^) | | | |
| --- | --- | --- | --- | --- | --- | --- | --- | --- | --- | --- | --- | --- | --- | --- | --- | --- | --- |
|  | | | | B | 95% CI | | p-value |  | B | 95% CI | | p-value |  | B | 95% CI | | p-value |
|  | | | |  | Lower | Upper |  |  |  | Lower | Upper |  |  |  | Lower | Upper |  |
| Birth |  | | |  |  |  |  |  |  |  |  |  |  |  |  |  |  |
| Mild or Moderate | | | | -0.23 | -0.72 | 0.26 | 0.349 |  | -0.10 | -0.29 | 0.08 | 0.273 |  | -0.24 | -0.60 | 0.13 | 0.200 |
| Severe | | | | -0.83 | -1.62 | -0.04 | **0.041** |  | -0.24 | -0.53 | 0.05 | 0.112 |  | -0.41 | -0.97 | 0.16 | 0.157 |
| 6 months | |  | |  |  |  |  |  |  |  |  |  |  |  |  |  |  |
| Mild or Moderate | | | | -0.22 | -0.71 | 0.28 | 0.393 |  | -0.10 | -0.28 | 0.09 | 0.315 |  | -0.20 | -0.54 | 0.14 | 0.246 |
| Severe | | | | -0.83 | -1.63 | -0.03 | **0.042** |  | -0.21 | -0.50 | 0.08 | 0.153 |  | -0.28 | -0.80 | 0.24 | 0.294 |
| 1 year |  | | |  |  |  |  |  |  |  |  |  |  |  |  |  |  |
| Mild or Moderate | | | | -0.20 | -0.74 | 0.34 | 0.472 |  | -0.09 | -0.29 | 0.12 | 0.399 |  | -0.16 | -0.48 | 0.15 | 0.315 |
| Severe | | | | -0.83 | -1.68 | 0.03 | 0.057 |  | -0.19 | -0.51 | 0.13 | 0.241 |  | -0.15 | -0.64 | 0.34 | 0.542 |
| 18 months | | |  |  |  |  |  |  |  |  |  |  |  |  |  |  |  |
| Mild or Moderate | | | | -0.18 | -0.79 | 0.43 | 0.566 |  | -0.08 | -0.32 | 0.16 | 0.502 |  | -0.12 | -0.43 | 0.18 | 0.418 |
| Severe | | | | -0.83 | -1.78 | 0.13 | 0.089 |  | -0.17 | -0.53 | 0.19 | 0.367 |  | -0.02 | -0.49 | 0.44 | 0.918 |
| 2 years |  | | |  |  |  |  |  |  |  |  |  |  |  |  |  |  |
| Mild or Moderate | | | | -0.16 | -0.86 | 0.54 | 0.655 |  | -0.07 | -0.35 | 0.20 | 0.602 |  | -0.09 | -0.38 | 0.21 | 0.561 |
| Severe | | | | -0.83 | -1.91 | 0.26 | 0.136 |  | -0.14 | -0.56 | 0.27 | 0.503 |  | 0.10 | -0.35 | 0.56 | 0.656 |
| 3 years |  | | |  |  |  |  |  |  |  |  |  |  |  |  |  |  |
| Mild or Moderate | | | | -0.12 | -1.05 | 0.80 | 0.794 |  | -0.06 | -0.43 | 0.31 | 0.756 |  | -0.01 | -0.31 | 0.29 | 0.936 |
| Severe | | | | -0.82 | -2.23 | 0.58 | 0.251 |  | -0.10 | -0.65 | 0.46 | 0.733 |  | 0.36 | -0.11 | 0.83 | 0.133 |
| 4 years |  | | |  |  |  |  |  |  |  |  |  |  |  |  |  |  |
| Mild or Moderate | | | | -0.09 | -1.26 | 1.09 | 0.885 |  | -0.04 | -0.51 | 0.43 | 0.856 |  | 0.06 | -0.27 | 0.40 | 0.715 |
| Severe | | | | -0.82 | -2.59 | 0.95 | 0.363 |  | -0.05 | -0.76 | 0.66 | 0.890 |  | 0.62 | 0.09 | 1.14 | **0.022** |
| 5 years |  | | |  |  |  |  |  |  |  |  |  |  |  |  |  |  |
| Mild or Moderate | | | | -0.05 | -1.48 | 1.38 | 0.946 |  | -0.03 | -0.61 | 0.55 | 0.922 |  | 0.14 | -0.25 | 0.53 | 0.491 |
| Severe | | | | -0.82 | -2.98 | 1.34 | 0.456 |  | -0.00 | -0.87 | 0.86 | 0.994 |  | 0.87 | 0.25 | 1.49 | **0.006** |

Supplementary Table 2: Associations between parasite density and height, weight, and BMI at specified ages in three adjusted^1^ linear mixed models.

1. Adjusted for residence during pregnancy, maternal pre-pregnancy BMI, maternal height, maternal education, sex, and gravidity. A polynomial spline captures the non-linear trend in height with respect to age. Random intercepts determined by individuals and mothers. Random slopes determined by repeated measurements of individuals. Perinatal mortalities excluded. Reference group is no detected parasites.

N = 301 for all models. Estimates (B) and 95% confidence intervals (CI) from adjusted linear mixed models. p < 0.05 bolded.
