# Supplementary material for: Association between placental malaria, postnatal linear growth, and body mass index in the Dogon Longitudinal Study, Mali
Source: Malar J. 2026 Jan 7;25:78. doi: 10.1186/s12936-025-05776-x (PMC12870135; doi:10.1186/s12936-025-05776-x)
Supplement: Supplementary file 5 — Supplementary material 5. Table of the 95% confidence intervals, point estimates, and p-values of association between parasite density and BMIat yearly intervals from birth to age five years adjusting for birth weight and birth BMI. [file 12936_2025_5776_MOESM5_ESM.docx]

Supplementary Table 5: Associations between parasite density and BMI (kg/m^2^) from birth to age 5 years in two adjusted^1^ linear mixed models.

|  | | | | Controlling for birth BMI | | | |  | Controlling for birth weight | | | |
| --- | --- | --- | --- | --- | --- | --- | --- | --- | --- | --- | --- | --- |
|  | | | | B | 95% CI | | p-value |  | B | 95% CI | | p-value |
|  | | | |  | Lower | Upper |  |  |  | Lower | Upper |  |
| Birth |  | | |  |  |  |  |  |  |  |  |  |
| Mild or Moderate | | | | -0.06 | -0.37 | 0.26 | 0.732 |  | -0.04 | -0.35 | 0.28 | 0.827 |
| Severe | | | | -0.12 | -0.62 | 0.38 | 0.648 |  | 0.04 | -0.46 | 0.54 | 0.878 |
| 6 months | |  | |  |  |  |  |  |  |  |  |  |
| Mild or Moderate | | | | -0.02 | -0.32 | 0.28 | 0.888 |  | -0.00 | -0.30 | 0.30 | 0.997 |
| Severe | | | | 0.03 | -0.45 | 0.48 | 0.958 |  | 0.17 | -0.30 | 0.64 | 0.476 |
| 1 year |  | | |  |  |  |  |  |  |  |  |  |
| Mild or Moderate | | | | 0.01 | -0.27 | 0.30 | 0.930 |  | 0.03 | -0.25 | 0.32 | 0.813 |
| Severe | | | | 0.14 | -0.30 | 0.58 | 0.530 |  | 0.30 | -0.14 | 0.75 | 0.184 |
| 18 months | | |  |  |  |  |  |  |  |  |  |  |
| Mild or Moderate | | | | 0.05 | -0.23 | 0.33 | 0.741 |  | 0.07 | -0.21 | 0.35 | 0.626 |
| Severe | | | | 0.27 | -0.16 | 0.70 | 0.218 |  | 0.43 | -0.00 | 0.87 | 0.051 |
| 2 years |  | | |  |  |  |  |  |  |  |  |  |
| Mild or Moderate | | | | 0.08 | -0.20 | 0.36 | 0.569 |  | 0.10 | -0.18 | 0.38 | 0.466 |
| Severe | | | | 0.40 | -0.03 | 0.83 | 0.070 |  | 0.56 | 0.13 | 1.00 | **0.011** |
| 3 years |  | | |  |  |  |  |  |  |  |  |  |
| Mild or Moderate | | | | 0.15 | -0.16 | 0.46 | 0.337 |  | 0.17 | -0.13 | 0.48 | 0.264 |
| Severe | | | | 0.66 | 0.18 | 1.13 | **0.007** |  | 0.82 | 0.35 | 1.30 | **0.001** |
| 4 years |  | | |  |  |  |  |  |  |  |  |  |
| Mild or Moderate | | | | 0.22 | -0.14 | 0.57 | 0.229 |  | 0.24 | -0.11 | 0.60 | 0.179 |
| Severe | | | | 0.92 | 0.36 | 1.47 | **0.001** |  | 1.09 | 0.53 | 1.65 | **>0.001** |
| 5 years |  | | |  |  |  |  |  |  |  |  |  |
| Mild or Moderate | | | | 0.29 | -0.13 | 0.71 | 0.182 |  | 0.31 | -0.11 | 0.74 | 0.145 |
| Severe | | | | 1.17 | 0.51 | 1.84 | **>0.001** |  | 1.35 | 0.68 | 2.01 | **>0.001** |

1. Adjusted for residence during pregnancy, maternal pre-pregnancy BMI, maternal height, maternal education, gravidity, and either birth BMI or birth weight. A polynomial spline captures the non-linear trend in BMI with respect to age. Random intercepts determined by individuals and mothers. Random slopes determined by repeated measurements of individuals. Perinatal mortalities excluded. Reference group is no detected parasites.

N = 301 for both models. Estimates (B) and 95% confidence intervals (CI) from adjusted linear mixed models. p < 0.05 bolded.
